# Anisotropic Local Structure of SrFe_2−*x*_Ni_*x*_As_2_ (x = 0.00, 0.16, and 0.23) Superconductor Probed by Polarized X-ray Absorption Fine Structure Measurements

**DOI:** 10.3390/ma17061301

**Published:** 2024-03-11

**Authors:** M. Y. Hacisalihoglu, L. Tortora, G. Tomassucci, L. Simonelli, N. L. Saini

**Affiliations:** 1 Department of Physics, Sapienza University of Rome, P. le Aldo Moro 2, 00185 Rome, Italy; muammeryasin.hacisalihoglu@uniroma1.it (M.Y.H.); lorenzo.tortora@uniroma1.it (L.T.); giovanni.tomassucci@uniroma1.it (G.T.); 2 Department of Physics, Recep Tayyip Erdogan University, Rize 53100, Turkey; 3 CELLS—ALBA Synchrotron Radiation Facility, Carrer de la Llum 2–26, Cerdanyola del Valles, 08290 Barcelona, Spain; lsimonelli@cells.es

**Keywords:** iron-based superconductors, local structure, X-ray absorption spectroscopy

## Abstract

We have investigated the effect of the Ni substitution on the local structure and the valence electronic states of the ${\mathrm{SrFe}}_{2-x}$${\mathrm{Ni}}_{x}$${\mathrm{As}}_{2}$ (x = 0.00, 0.16, and 0.23) superconductor with a multi-edge extended X-ray absorption fine structure (EXAFS) and X-ray absorption near edge structure (XANES) spectroscopy. The As K-edge and Fe K-edge EXAFS measurements in the two polarizations (E‖ab and E‖c) show a clear change in the local structure with Ni concentration. The near-neighbor bondlengths and the related mean-square relative displacements (MSRDs) decrease as the Ni content increases. The polarized XANES spectra at the As, Fe and Ni K edges reveal a systematic change in the anisotropy of the valence electronic structure. The results suggest that the quasi 2D electronic structure of this system tends to become more isotropic as the Ni content increases. The local structure and the valence electronic states are discussed in the frame of the evolving electronic transport of the ${\mathrm{SrFe}}_{2-x}$${\mathrm{Ni}}_{x}$${\mathrm{As}}_{2}$ system.

## 1. Introduction

The discovery of superconductivity in oxygen-free ternary pnictide ${\mathrm{BaFe}}_{2}$${\mathrm{As}}_{2}$ (122) with $T_{c}$∼38 K [1] has stimulated much interest, and several 122-type superconducting materials have been found [2,3,4,5,6] with ${\mathrm{AEFe}}_{2}$${\mathrm{As}}_{2}$ (AE = Ba, Sr, Ca, and Eu) being the parent compounds [7,8,9,10,11]. Transition metal substitutions for Fe in this system are of significant interest, since such substitutions are known to stabilize the superconductivity [12]. Transition-metal doping with either holes [13,14,15,16,17] or electrons [6,18,19,20,21,22,23,24,25] suppresses the spin density wave (SDW) in the parent 122 system with the appearance of electron-doped superconductivity [26].

The transition-metal-doped and iron-based superconductors show similar onset concentrations; however, the two-electron-doped systems are characterized by lower optimal $T_{c}$ than those doped by one electron [6]. For example, the Nickel-doped system (two-itinerant doping) shows a phase diagram, which appears with a narrow and strongly suppressed superconducting dome than the most-studied Cobalt-doped system (one-electron doping) [6,16]. This behaviour of Ni doping has been observed in different 122 systems, including ${\mathrm{BaFe}}_{2}$${\mathrm{As}}_{2}$ (Ba122) [20,27], ${\mathrm{SrFe}}_{2}$${\mathrm{As}}_{2}$ (Sr122) [6], and ${\mathrm{CaFe}}_{2}$${\mathrm{As}}_{2}$ (Ca122) [24,28]. Looking at the phase diagram of ${\mathrm{SrFe}}_{2-x}$${\mathrm{Ni}}_{x}$${\mathrm{As}}_{2}$, the superconductivity is observed in the Ni concentration range of 0.10 ≤ x ≤ 0.22, with the highest $T_{c}$ of 9.8 K for x∼0.16 [29]. The parent ${\mathrm{SrFe}}_{2}$${\mathrm{As}}_{2}$ is a quasi two-dimensional (2D) paramagnetic metal that crystallizes in a ${\mathrm{ThCr}}_{2}$${\mathrm{Si}}_{2}$ (space group $I_{4mmm}$)-type tetragonal structure and undergoes a structural phase transition from a tetragonal to orthorhombic (space group $F_{mmm}$), accompanied by a spin-density-wave (SDW) ordering of Fe spins [9,29,30,31] below a temperature $T_{N,S}$∼ of 205 K. The $T_{N,S}$ is sensitive to the sample quality; thus, it can vary from sample to sample [16].

Similar to other emerging superconductors, the phase diagram of the iron-based superconductors is characterized by a complex interplay between different electronic degrees of freedom including charge, spin and the underlying lattice [32]. Such a complexity is associated to the fact that these materials are multiband electronic systems in which six electrons occupy five iron orbitals. Therefore, the electronic transport is highly susceptible to any small disorder and atomic displacements [33]. For example, a superconductivity with $T_{c}$ of ∼21 K has been induced without doping in the parent ${\mathrm{SrFe}}_{2}$${\mathrm{As}}_{2}$ compound by a lattice strain [34]. It has been shown that the strain-induced superconductivity in ${\mathrm{SrFe}}_{2}$${\mathrm{As}}_{2}$ is tunable through heat treatment and mechanical deformation [34]. Such studies are a mere indication of the structural susceptibility of ${\mathrm{SrFe}}_{2}$${\mathrm{As}}_{2}$ in which the local structure and disorder may have some important consequences on the electronic transport, and hence on the superconductivity in ${\mathrm{SrFe}}_{2-x}$${\mathrm{Ni}}_{x}$${\mathrm{As}}_{2}$. Furthermore, ab initio theoretical calculations on the iron-based superconductors have revealed that the Fe ${3d}_{x^{2}-y^{2}}$ orbital occupation has a strong dependence on local structure parameters such as the z-position of As atoms, largely affecting the Fe magnetism [30].

In this work, we have investigated the local structure of ${\mathrm{SrFe}}_{2-x}$${\mathrm{Ni}}_{x}$${\mathrm{As}}_{2}$ as a function of the Ni substitution by the site-specific X-ray absorption spectroscopy. In particular, As K-edge (∼11,867 eV) and Fe K-edge (∼7112 eV)-extended X-ray absorption fine structure (EXAFS) measurements in E‖ab and E‖c polarization geometries are used to probe the local structure around the As and and Fe atoms in the structure. This is combined with the polarized As K-edge, Fe K-edge and Ni K-edge (∼8333 eV) X-ray absorption near the edge structure (XANES) analysis to probe the local geometry and the valence electronic structure of ${\mathrm{SrFe}}_{2-x}$${\mathrm{Ni}}_{x}$${\mathrm{As}}_{2}$. The EXAFS results show a small but systematic evolution of the local structure with the Ni substitution. The XANES results reveal a clear anisotropy of the valence electronic structure that changes substantially with the Ni concentration. The results are discussed in the frame of a changing electronic transport of the title system as a function of Ni doping.

## 2. Materials and Methods

We performed X-ray absorption measurements on well-characterized single crystals of ${\mathrm{SrFe}}_{2-x}$${\mathrm{Ni}}_{x}$${\mathrm{As}}_{2}$ (x = 0.00, 0.16, and 0.23) with the surface area of 1–3 ${\mathrm{mm}}^{2}$ [6]. X-ray absorption measurements were carried out on the beamline BM23 at the European Synchrotron Radiation Facility (ESRF). The X-ray beam at the BM23 of the 6-GeV synchrotron storage ring was monochromatized by a Si(311) double crystal monochromator. The beam was sagittally focused on the single crystal samples mounted in a continuous flow He cryostat. All measurements were performed at 20 K and maintained within ±1 K during the data collection. Two different polarization geometries were used for the measurements; the normal incidence geometry (E‖ab) in which the electric vector of the linearly polarized X-ray beam is parallel to the ab-plane (α⋍10°) and the grazing incidence geometry (E‖c) where the polarization is almost perpendicular to the ab-plane (α⋍80°). The X-ray absorption spectra on ${\mathrm{SrFe}}_{2-x}$${\mathrm{Ni}}_{x}$${\mathrm{As}}_{2}$ crystals were recorded in both geometries at the Fe K-edge (∼7112 eV), As K-edge (∼11,867 eV), and Ni K-edge (∼8333 eV), collecting the $K_{\alpha }$ fluorescence photons of respective elements using a multi-element Ge-pixel detector system. Sequential measurements on the three samples mounted with a reference were performed at respective edges. In order to ensure the spectral reproducibility, multiple absorption scans were recorded. The Fe K-edge measurements on the same samples were repeated at the CLAESSbeamline of the 3-GeV ALBA synchrotron radiation facility in Cerdanyola del Valles (Barcelona). At the CLAESS, the synchrotron radiation is emitted by a multipole wiggler source with the Si(111) monochromator being used for the measurements. These measurements were carried out at several temperatures in the E‖ab geometry. The EXAFS oscillations from the X-ray absorption data were extracted using a standard procedure based on a polynomial spline function fit to the pre-edge subtracted spectra [35], followed by a correction of the EXAFS signal for the X-ray fluorescence self-absorption using the algorithm developed by Tröger et al. [36]. The atomic absorption, determined via a linear fit far from the absorption edge jump, was used to normalize the XANES spectra.

## 3. Results and Discussion

### 3.1. Extended X-ray Absorption Fine Structure (EXAFS) Analysis

**Figure 1:**
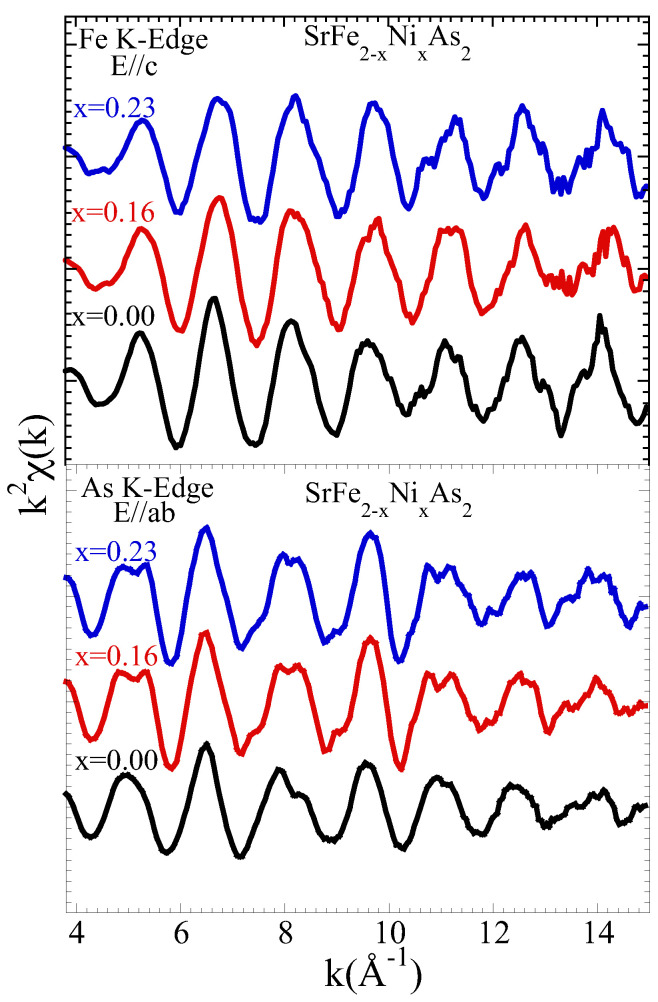
EXAFS oscillations (weighted by $k^{2}$) extracted from X-ray absorption measurements at Fe K-edge (**upper panel**) in the E‖c geometry and As K-edge (**lower panel**) in the E‖ab geometry measured on ${\mathrm{SrFe}}_{2-x}$${\mathrm{Ni}}_{x}$${\mathrm{As}}_{2}$ (x = 0.00, 0.16, and 0.23) crystals at 20 K. The spectra are shifted artificially for better visualization.

We start our discussion by presenting EXAFS results. It is worth recalling that EXAFS is a site-selective experimental technique to probe the local structure [35]. In the X-ray absorption process, photoelectron expelled from the selected atom travels in the system and backscatters from the surrounding atoms. The outgoing electron wave interferes with the backscattered electron wave, and this produces oscillations in the X-ray absorption coefficient measured as a function of energy (wavevector). The information on the local structure is contained in these oscillations. Using a polarized X-ray beam the directional information on the local structure can be obtained. The layered crystal structure of ${\mathrm{SrFe}}_{2}$${\mathrm{As}}_{2}$ is characterized by the electronically active ${\mathrm{FeAs}}_{4}$ layers sandwiched between the Sr layers [31]. Here, the focus is on the Fe-As atomic correlations, and hence the E‖c geometry has been used for the Fe K-edge measurements to separate Fe-As from the Fe-Fe bond contribution. Similarly, information on the same Fe-As bond is also available from the As K-edge EXAFS for which E‖ab geometry has been used. Figure 1 shows EXAFS oscillations (multiplied by $k^{2}$) as a function of the photoelectron wave vector *k*, extracted from the X-ray absorption measurements at the Fe K-edge in the E‖c geometry and the As K-edge in the E‖ab geometry on ${\mathrm{SrFe}}_{2-x}$${\mathrm{Ni}}_{x}$${\mathrm{As}}_{2}$ (x = 0.00, 0.16, and 0.23). The oscillations for both edges are clearly visible up to a *k*∼15 ${Å}^{-1}$. Although small, some evident changes can be observed in As K- (see, e.g., k-range ∼4–6, ∼8–10 ${Å}^{-1}$) and Fe K-edges (see, e.g., 5–8 ${Å}^{-1}$) EXAFS oscillations revealing differing local structure of the three samples containing a different Ni concentration. The changes are apparent in As K-edge and Fe K-edge EXAFS oscillations.

**Figure 2:**
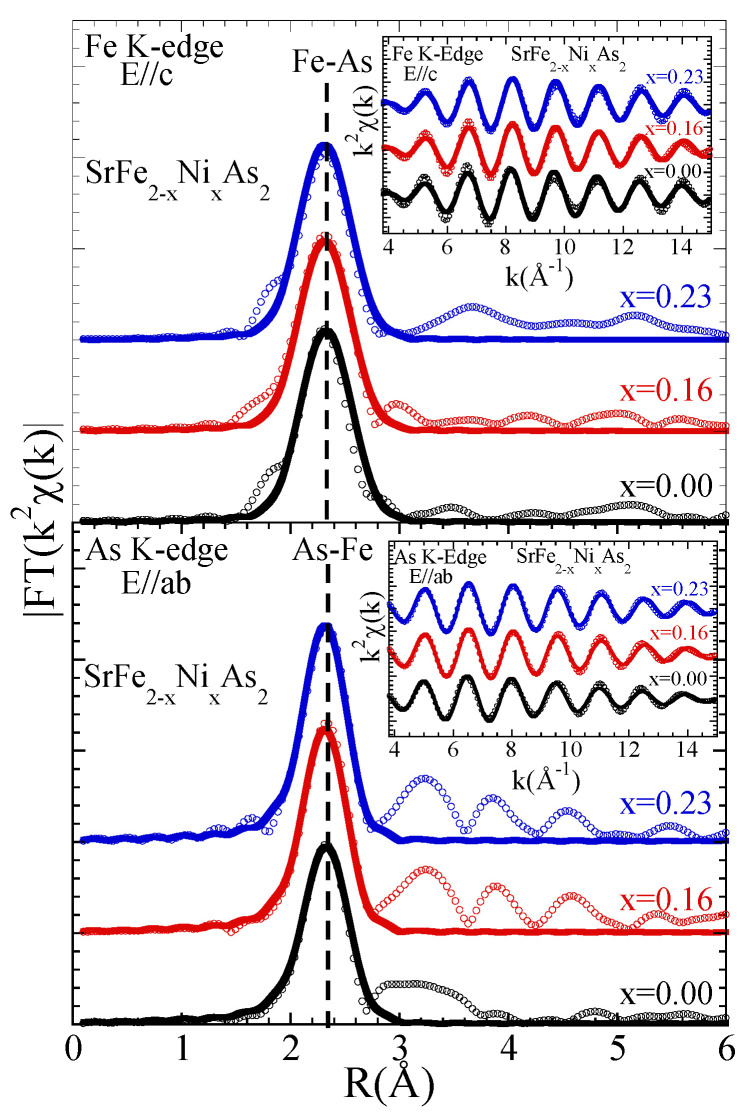
Fourier transform (FT) magnitudes of the EXAFS oscillations (Figure 1). The FTs are performed using Gaussian windows with the k-range of 3.8–15 ${Å}^{-1}$ for both the As K- and Fe K-edge EXAFS oscillations. Model fits to the FTs are also shown as solid lines. The insets show the filtered EXAFS oscillations (symbols) together with the *k*-space model fits (solid line).

The above differences are clearly reflected in the real space that can be observed in the Fourier Transforms (FTs) of the EXAFS oscillations. The FT magnitudes are shown in Figure 2. The FTs are performed using Gaussian windows with the k-range of 3.8–15 ${Å}^{-1}$ for the As K-edge as well as for the Fe K-edge EXAFS oscillations. The main peak (at ∼2.3 Å) in the As K-edge FTs magnitude is only due to the As-Fe bondlength. The magnitude of the main peak gradually increases with a small but an apparent change in its position with the Ni substitution. A gradual change in the As-Fe bondlength could be the cause of the changes in the magnitude. Similarly, the main peak in the Fe K-edge (at ∼2.3 Å) is due to the Fe-As bondlength. Again, the main peak seems to evolve in position, similar to the As K-edge; however, no apparent change can be observed in its amplitude. The changing local structure due to the Ni substitution can also be observed from the FTs’ peak structures at R ≳ 2.8 Å, which are due to longer distance contributions mixed with multiple scatterings. In particular, there is an eye-catching change in the FT feature at ∼3–4 Å in both edges. In the FTs of the As K-edge EXAFS, measured in the E‖ab geometry, the feature is due to As-As distances, including contributions from the in-plane As atoms mixed with the As-Sr scattering [37]. On the other hand, in the FTs of the Fe K-edge, measured in the E‖c geometry, the feature should be mainly due to Fe-Sr scattering. In both cases, the magnitude of these features increase, which is a mere indication that an average configurational disorder due to the Ni substitution is decreasing.

The changes with the Ni substitution can be quantified through the local structure parameters, which are determined by the EXAFS model fits. For this purpose, we have used the conventional approach based on single-scattering approximation [35], in which the EXAFS oscillations at two different edges were modelled by the standard EXAFS equation [35]:

$$\chi \left(k\right)=\sum_{i}\frac{N_{i}S_{0}^{2}}{kR_{i}^{2}}f_{i}\left(k,R_{i}\right)e^{\frac{-2R_{i}}{\lambda }}e^{-2k^{2}\sigma_{i}^{2}}sin\left[2kR_{i}+\delta_{i}\left(k\right)\right]$$

where $N_{i}$ is the number of near-neighbor atoms at a distance $R_{i}$ from the photoabsorbing As and Fe atoms. The backscattering amplitude, the phase shifts and the photoelectron mean free path are $f_{i}\left(k,R_{i}\right)$, $\delta_{i}$, and λ, respectively. The correlated Debye–Waller factor (DWF) parameter, measuring the mean square relative displacement (MSRD) of the photoabsorber-backscatter pairs, is denoted as $\sigma_{i}^{2}$. The EXAFS amplitude suffers from a reduction due to many body effects while the photoelectron propagates in the matter (plasmons, electron-hole pairs, excitations, etc.). There are intrinsic losses due to shake-up and shake-off excitations related with the core-hole in the X-ray absorption process. The scale factor $S_{0}^{2}$ takes into account all of these losses in the EXAFS equation. The least-squares fits were performed with the average structure of ${\mathrm{SrFe}}_{2}$${\mathrm{As}}_{2}$ [31] being the starting model. The crystal structure contains four Fe atoms (at a distance ∼2.39 Å) and four As atoms (at a distance ∼2.39 Å); this is observed in the As K- and Fe K-edges, respectively. This enables us to directly observe the effect of the Ni substitution only on the Fe-As bond measured from the As site (As K-edge) and from the Fe site (Fe K-edge). The effect of beam polarization (since single crystal samples are used) on the $N_{i}$ has been accounted using an effective number of neighbors that considers projections of the bond with respect to the X-ray beam polarization vector. The EXCURVE 9.275 code (with calculated backscattering amplitudes and phase shift functions) [38] was used for the model fits [35].

The scale factor due to the passive electron reduction factor, $S_{0}^{2}$, was fixed to 1.0 (consistent with earlier EXAFS studies) and the photoelectron energy zero ($E_{0}$) was fixed after fit trials on different scans and the reference sample. Therefore, only the near-neighbor distance $R_{i}$ and the corresponding $\sigma_{i}^{2}$ (i.e., the DWF parameter), were the free parameters in the model fits. The model fits in the real space are shown as solid lines in Figure 2, while the *k*-space model fits (solid line) are shown as insets including the filtered EXAFS oscillations. The number of independent data points, $N_{\mathrm{ind}}∼$(2ΔkΔR)/π [35], were about 9 (Δk = 11.2 ${Å}^{-1}$ and ΔR = 1.3 Å), with the number of free parameters being two for both edges.

**Figure 3:**
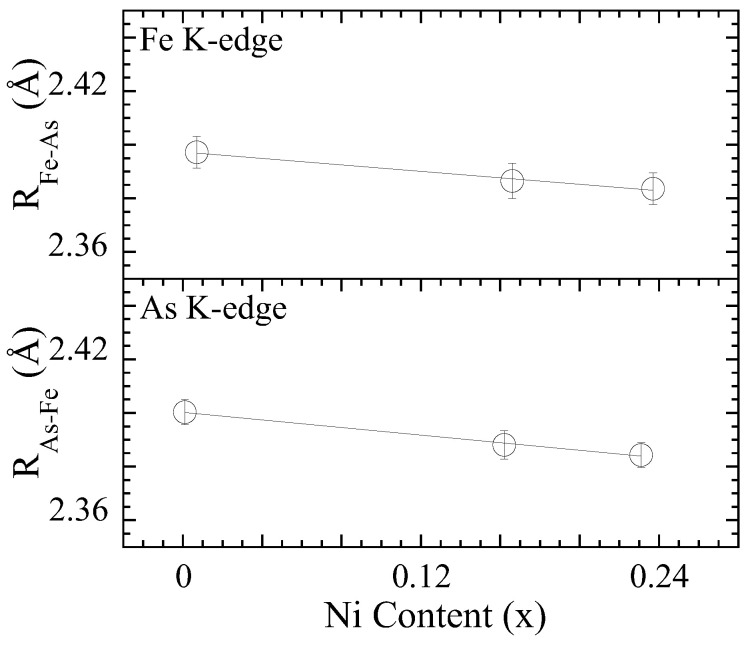
Local Fe-As bondlength as a function of Ni concentration determined by the Fe K-edge EXAFS in the E‖c geometry (**upper panel**) and the As K-edge EXAFS in the E‖ab geometry (**lower panel**). The error bars represent the uncertainties estimated by the best fits to five different EXAFS scans on the Fe and As K-edges.

Figure 3 displays the Fe-As local bondlengths in ${\mathrm{SrFe}}_{2-x}$${\mathrm{Ni}}_{x}$${\mathrm{As}}_{2}$ as a function of the Ni concentration obtained by EXAFS model fits of As K-edge (lower panel) in the E‖ab polarization geometry and Fe K (upper panel)-edge in the E‖c geometry. The average As-Fe distance is found to be ∼2.402 Å for the parent compound and ∼2.387 Å and ∼2.386 Å for x = 0.16 and 0.23, respectively, from the As K-edge; the average Fe-As distance is found to be ∼2.398 Å for the parent compound and ∼2.386 Å and ∼2.384 Å for x = 0.16 and 0.23, respectively, from the Fe K-edge. The As-Fe distance, obtained from the E‖ab-polarized As K-edge EXAFS, appears slightly longer than the Fe-As distance obtained from the E‖c-polarized Fe K-edge EXAFS. This indicates anisotropic atomic displacements in ${\mathrm{SrFe}}_{2-x}$${\mathrm{Ni}}_{x}$${\mathrm{As}}_{2}$, including smaller fluctuations of the As atoms in the c axis than in the ab-plane.

The Fe-As bondlength, measured by the two edges, reveals a contraction with the Ni substitution. This should be related to the smaller Ni ion with respect to Fe entering the structure without introducing any configurational disorder. Indeed, the residual resistivity results of Saha et al. suggests that the Ni substitution for Fe introduces minimal disorder in the system, even up to x∼0.3 [6]. Furthermore, it should be mentioned that the Fe-As distance is highly covalent and hence large changes from the substitution are not expected; however, any small change may have a significant effect on the electronic structure of the system. Indeed, ab initio calculations on the parent compound reveal an increasing occupation of ${3d}_{x^{2}-y^{2}}$ orbitals with a decreasing Fe-As distance because of a downward shift of the related band near Γ [30].

**Figure 4:**
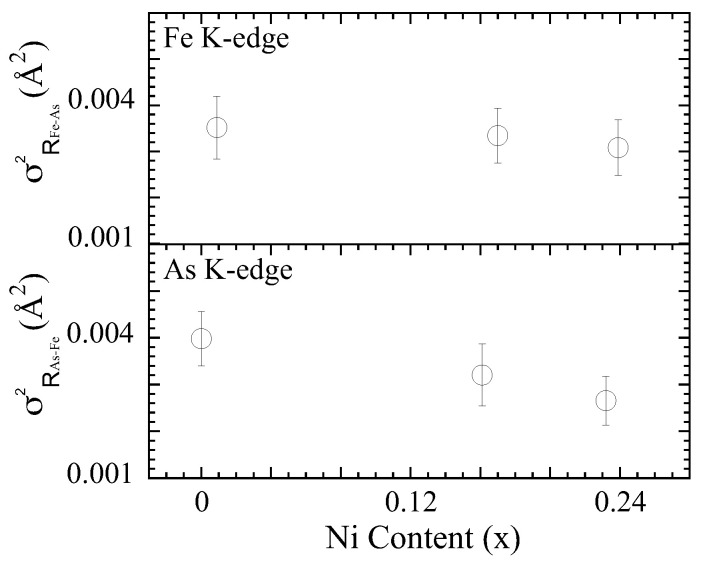
The mean square relative displacement parameter ($\sigma^{2}$) of the Fe-As bondlength as a function of Ni concentration, as determined by the Fe K-edge EXAFS in the E‖c geometry (**upper panel**) and the As K-edge EXAFS in the E‖ab geometry (**lower panel**).

The EXAFS Debye–Waller Factor parameter ($\sigma_{i}^{2}$), measuring the mean square relative displacements (MSRD) of the pair of atoms, (i.e., distance–distance correlation function) can provide further information on the atomic displacements in the system as a function of the Ni substitution. Figure 4 displays $\sigma^{2}$ of the Fe-As bondlengths obtained from the As K (lower panel) and the Fe K (upper panel)-edge EXAFS analysis as a function of the Ni concentration on ${\mathrm{SrFe}}_{2-x}$${\mathrm{Ni}}_{x}$${\mathrm{As}}_{2}$. The $\sigma^{2}$ of the As-Fe bondlengths (As K-edge) is found to be ∼0.00398 ${Å}^{2}$ for the parent compound that decreases to ∼0.00320 ${Å}^{2}$ and ∼0.00266 ${Å}^{2}$ for x = 0.16 and 0.23, respectively. Similarly, the $\sigma^{2}$ of the Fe-As bondlengths (Fe K-edge) is found to be ∼0.00352 ${Å}^{2}$ for the parent compound and ∼0.00334 ${Å}^{2}$ and ∼0.00308${Å}^{2}$ for x = 0.16 and 0.23, respectively. Although the $\sigma^{2}$ hardly show any change with the Ni substitution, there seems to be a small decreasing trend with the increasing Ni concentration, which is evidently observed from both As K-edge and Fe K-edge results. Apart from others, the $\sigma^{2}$ is an indicator of the configurational disorder in the respective bondlength. The fact that the $\sigma^{2}$ hardly shows any change (or tends to decrease) is an indicator that Ni atoms are likely entering the structure via substitution with a negligible (or decreased with respect to the x = 0.0) disorder in the lattice.

### 3.2. X-ray Absorption near Edge Structure (XANES) Analysis

**Figure 5:**
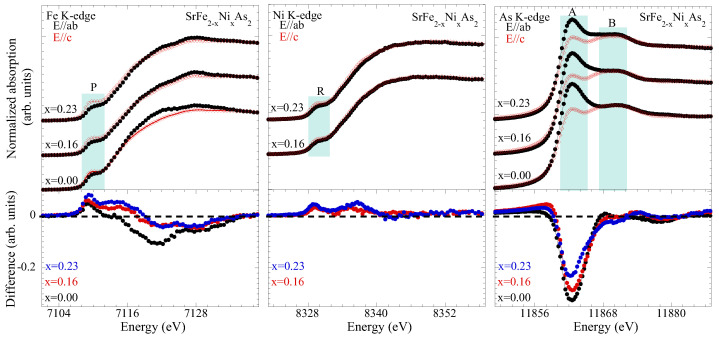
Normalized XANES spectra of ${\mathrm{SrFe}}_{2-x}$${\mathrm{Ni}}_{x}$${\mathrm{As}}_{2}$, measured in both E‖ab and E‖c geometries at the Fe K-edge (**upper-left panel**), the Ni K-edge (**upper-middle panel**), and the As K-edge (**upper-right panel**). The bottom panels depict the spectral differences at the Fe K-edge (**bottom-left panel**), Ni K-edge (**bottom-middle panel**), and As K-edge (**bottom-right panel**).

Let us move to discuss the XANES spectra of the title system. The XANES spectroscopy is a higher-order probe of atomic correlations providing information on the local geometry and the valence electronic states [35,39]. XANES is highly sensitive to any small atomic displacements that may not show up in the EXAFS. Here, we have exploited XANES spectroscopy to obtain further information, not only on the local geometry but also on the electronic valence states. The XANES spectra obtained at the Fe K-edge, As K-edges and Ni K-edge have been analyzed. The spectra are measured using two different experimental geometries: normal incidence geometry (in which the polarization of the X-ray beam is parallel to the ab-plane, i.e., E‖ab), and grazing incidence geometry (in which the polarization of the X-ray beam is almost parallel to the c-axis, i.e., E‖c). Figure 5 displays the normalized XANES spectra of ${\mathrm{SrFe}}_{2-x}$${\mathrm{Ni}}_{x}$${\mathrm{As}}_{2}$, which is measured at three absorption edges (the As K-, Fe K- and Ni K-edges) in E‖ab and E‖c geometries. The atomic absorption, determined via a linear fit far from the absorption edge jumps, was used to normalize the XANES spectra. The XANES spectral differences, which shows the difference between the grazing incidence spectra and the normal incidence spectra of the individual substitutions (E‖c-E‖ab), and reflects the geometrical and electronic anisotropy of ${\mathrm{SrFe}}_{2-x}$${\mathrm{Ni}}_{x}$${\mathrm{As}}_{2}$, are also included in the lower panels for different Ni substitutions.

**Figure 6:**
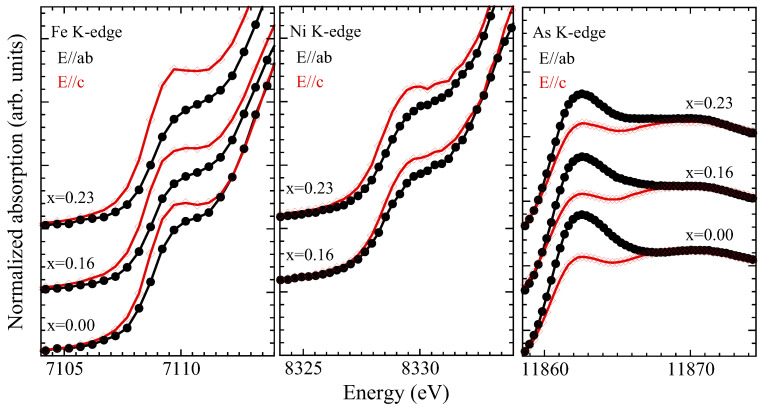
A zoom-over the XANES pre-peak feature P in the Fe K-edge (**left**) and feature R in the Ni K-edge (**center**) spectra. The As K-edge near-edge features A and B are also shown zoomed in (**right**).

We will start our discussion on the Fe K and Ni K-edge XANES spectra and their evolution with the Ni substitution. The ground-state electronic configurations of iron and nickel atoms are [Ar]$3d^{6}4s^{2}$ and [Ar]$3d^{8}4s^{2}$, respectively. The Fe(Ni) K-edge absorption process is mainly governed by the Fe (Ni) 1*s*→4*p* dipole transition in the continuum. In addition to the dipole, a direct quadrupole transition in the unoccupied Fe(Ni) 3*d* states is possible and can be observed as a weak pre-peak, denoted here as P (R) in the Fe K-edge (Ni K-edge) spectra. The pre-peak P (R) also contains dipole contribution due to local distortions [40,41], mixing d and p-symmetry orbitals. A zoom over the pre-peak feature P(R) is shown in Figure 6, which compares this feature to the two polarizations. Since the contribution of the direct quadrupole 1*s*→ 3*d* transition is expected to be small, the intense pre-peak P(R) results from the hybridizing orbitals [41,42,43] and, in particular, the intense pre-peak P(R) is mainly due to hybridized Fe (Ni) 3*d* and pnictogen (As) - 4*p* orbitals.

Looking at the Fe K-edge XANES spectra, we can make some clear observations: (i) the spectra in both polarizations shift towards higher energy with the increasing Ni substitution, albeit the shift for the E‖c spectra is smaller than that of E‖ab; (ii) the spectral weight of the continuum beyond ∼7116 eV is lower in the E‖c geometry; (iii) the spectral difference between the two polarizations is decreasing with the Ni substitution; (iv) the pre-peak in the E‖c geometry is more intense than the one in the E‖ab geometry (Figure 6). The X-ray absorption edge energy position is sensitive to the near-neighbor local geometry [35], and a positive (negative) energy shift of the XANES spectrum would mean shorter (longer) bondlengths. Therefore, the shift towards a higher energy of the XANES spectra indicates a decrease in near-neighbor distances. Since the nearest neighbors of Fe are As atoms, the observed shift is partly due to a contraction of Fe-As distances (Figure 3). The decreasing spectral difference with the Ni substitution in the continuum region is due to the fact that the energy shift of the spectra in the E‖ab and E‖c are different. The decreased spectral difference indicates that the structural anisotropy in the system is decreasing with the Ni concentration. Similar observations can be made from the Ni K-edge XANES; i.e., Ni is effectively substituting the Fe in the structure.

On the other hand, the valence electronic structure seems to suffer the substantial effect of the Ni substitution (Figure 6). Indeed, the pre-peak P(R), reflecting a direct transition in the unoccupied Fe (Ni) 3*d* and admixed As 4p orbitals, reveals a clear change in the electronic anisotropy. In the E‖c geometry, the 3$d_{xz,yz}$ and 3 $d_{z^{2}}$ orbitals can be reached in the transition process. Instead, the 3$d_{xz,yz}$ are reached in the E‖ab geometry. Therefore, the lower intensity of the pre-peak in the E‖ab geometry is not surprising. However, it is interesting to observe that the electronic structure anisotropy, determined by the difference in the pre-peak in the two polarizations, is substantially affected by the Ni substitution. This trend is similar in the Fe K-edge XANES as well as in the Ni K-edge XANES. This indicates that the electronic structure of Fe and Ni is similar, since Ni enters the ${\mathrm{FeAs}}_{4}$ lattice, i.e., the 3*d*–4*p* hybridization in the two cases should be qualitatively the same. Therefore, the evolution of the anisotropy is governed by the hybridization of the Fe (Ni) 3*d* and pnictogen As - 4*p* orbitals. Further information on this electronic anisotropy can be obtained from the As K-edge XANES spectra, in which a direct As 1*s*→4*p* transition is probed.

Figure 5 (right) displays the As K-edge XANES spectra of ${\mathrm{SrFe}}_{2-x}$${\mathrm{Ni}}_{x}$${\mathrm{As}}_{2}$ for three Ni concentrations. As above, the As K-edge spectra in the two polarization geometries are shown for different Ni substitutions. The ground-state electronic configuration of As is [Ar]$3d^{10}4s^{2}\;4p^{3}$, and the As K-edge spectra probe the transition from the As 1*s* core-level into the unoccupied 4*p* and admixed electronic states. The As K-edge XANES spectra are characterized by the main peak feature, denoted as A, and the continuum resonances appearing as a broad structure, at ∼6 eV above the edge jump, which is denoted by B. The peak feature A is described by the dipole transition from As 1*s* core-level electrons to the unoccupied As 4*p* states admixed with Fe (Ni) 3*d* orbitals. Multiple scattering calculations are commonly used for the interpretation of different XANES features [35]. In the present case, we have used already known (see, e.g., [44]) multiple scattering calculations to discuss different features of the As K-edge XANES spectra measured in ${\mathrm{SrFe}}_{2-x}$${\mathrm{Ni}}_{x}$${\mathrm{As}}_{2}$.

The XANES feature A is more intense in the E‖ab geometry, indicating that the unoccupied As 4*p* states near the Fermi level are primarily derived by the $p_{xy}$ orbital symmetry. This is also understandable due to the fact that the electronic density of states near the Fermi level is mainly driven by the 3$d_{xz,yz}$ states that become admixed with As $4p_{xy}$ orbitals in the Fe-As bonding. Therefore, the feature A in the E‖ab geometry is more intense than the one in the E‖c geometry, probing the unoccupied $4p_{z}$ orbitals (Figure 6). The As $4p_{z}$ orbitals can be admixed also with Fe (Ni) 3$d_{z^{2}}$ orbitals, and hence there is a higher intensity of the pre-peak P(R) in the Fe(Ni) K-edge spectra measured with E‖c polarization. The feature A in the As K-edge XANES exhibits significant dependence on polarization and on the Ni substitution (Figure 6). Indeed, this feature in the E‖ab geometry shows a continuous decrease; in the E‖c geometry, it tends to increase with the increasing Ni substitution. It is worth recalling that the pre-peak P(R) of Fe (Ni) K-edge reveals increased Fe(Ni) 3*d*-orbital anisotropy with the Ni substitution, while the pre-peak A of As K-edge shows that the As 4*p* orbital anisotropy is decreasing. This apparently contradicting behavior is due to the fact that Fe 3*d* and As 4*p* orbitals are strongly hybridized in the Fe-As bonding, with the ${\mathrm{FeAs}}_{4}$ layer getting thicker with the Ni substitution and hence causing a reduced structural anisotropy.

## 4. Conclusions

In summary, we studied the local structure and valence electronic structure of Ni doped 122 superconductor by the polarized As K-edge, Fe K-edge, and Ni K-edge EXAFS and XANES measurements. The Fe K-edge (As K-edge) EXAFS reveals that the Fe-As (As-Fe) distance decreases with the Ni substitution, while hardly showing any change in the related mean square relative displacements. Polarized XANES spectra show clear orbital anisotropy that suffers a significant change with the Ni substitution. The Fe K-edge and Ni K-edge XANES spectra show a clear and similar change in the electronic anisotropy, with the Ni substitution suggesting that the Ni atoms are occupying the Fe sites in the lattice. The electronic anisotropy is further revealed by the As K-edge XANES spectra showing a decreasing anisotropy with increasing Ni. The polarized XANES spectra suggest that the quasi 2D electronic structure of this system becomes more isotropic with the increasing Ni concentration. In conclusion, the results provide further information on the anisotropic local atomic displacements that should have a direct implication on the spin/orbital fluctuation models [45,46] for the electronic transport in these materials.

## Data Availability

Data can be obtained by a reasonable request from the corresponding author.
